# Assessing the Accuracy of Artificial Intelligence Models in Scoliosis Classification and Suggested Therapeutic Approaches

**DOI:** 10.3390/jcm13144013

**Published:** 2024-07-09

**Authors:** Artur Fabijan, Agnieszka Zawadzka-Fabijan, Robert Fabijan, Krzysztof Zakrzewski, Emilia Nowosławska, Bartosz Polis

**Affiliations:** 1 Department of Neurosurgery, Polish-Mother’s Memorial Hospital Research Institute, 93-338 Lodz, Poland; krzysztof.zakrzewski@iczmp.edu.pl (K.Z.); emilia.nowoslawska@iczmp.edu.pl (E.N.); jezza@post.pl (B.P.); 2 Department of Rehabilitation Medicine, Faculty of Health Sciences, Medical University of Lodz, 90-419 Lodz, Poland; agnieszka.zawadzka@umed.lodz.pl; 3 Independent Researcher, Luton LU2 0GS, UK; robert.f.fabijan@gmail.com

**Keywords:** scoliosis, artificial intelligence, PMC-LLaMA, ChatGPT 4, clinical decision support systems

## Abstract

**Background:** Open-source artificial intelligence models (OSAIMs) are increasingly being applied in various fields, including IT and medicine, offering promising solutions for diagnostic and therapeutic interventions. In response to the growing interest in AI for clinical diagnostics, we evaluated several OSAIMs—such as ChatGPT 4, Microsoft Copilot, Gemini, PopAi, You Chat, Claude, and the specialized PMC-LLaMA 13B—assessing their abilities to classify scoliosis severity and recommend treatments based on radiological descriptions from AP radiographs. **Methods:** Our study employed a two-stage methodology, where descriptions of single-curve scoliosis were analyzed by AI models following their evaluation by two independent neurosurgeons. Statistical analysis involved the Shapiro–Wilk test for normality, with non-normal distributions described using medians and interquartile ranges. Inter-rater reliability was assessed using Fleiss’ kappa, and performance metrics, like accuracy, sensitivity, specificity, and F1 scores, were used to evaluate the AI systems’ classification accuracy. **Results:** The analysis indicated that although some AI systems, like ChatGPT 4, Copilot, and PopAi, accurately reflected the recommended Cobb angle ranges for disease severity and treatment, others, such as Gemini and Claude, required further calibration. Particularly, PMC-LLaMA 13B expanded the classification range for moderate scoliosis, potentially influencing clinical decisions and delaying interventions. **Conclusions:** These findings highlight the need for the continuous refinement of AI models to enhance their clinical applicability.

## 1. Introduction

In the past year, there has been increased interest in the use of open-source artificial intelligence models (OSAIMs) in medical support contexts [1]. Data concerning scoliosis are increasingly being utilized in artificial intelligence (AI) research aimed at diagnostic and therapeutic supports [2]. Since the launch of the fourth-generation GPT model by OpenAI, the scientific publishing sector has experienced a significant surge in interest. Research is particularly focused on the potential of ChatGPT 4 in addressing medical challenges. The model’s ability to solve medical test problems [3], provide educational support to students [4] and patients [5], and its applications as diagnostic and therapeutic tools are under scrutiny [6,7].

**Figure 1:**
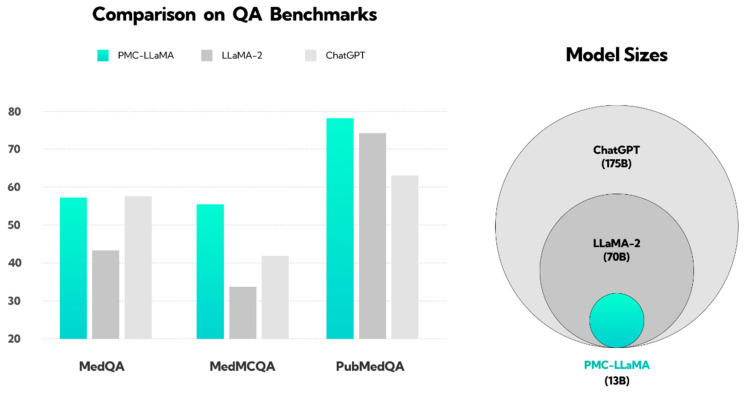
Illustration showing the performance of the PMC-LLaMA model compared to ChatGPT and LLaMA-2 in response to medical questions. This figure has been reconstructed based on the results from the work of Wu et al. [22]. The PubMedQA dataset consists of biomedical questions and answers collected from PubMed abstracts. The MedMCQA dataset comprises multiple-choice questions sourced from mock and past exams of two Indian medical school entrance tests, AIIMS and NEET-PG. The MedQA dataset features questions derived from medical exams. The y-axis represents the QA benchmark score; question answering (QA). The pie chart on the right illustrates the size of the language models, with ChatGPT (light gray) trained on the largest dataset, followed by LLaMA-2 (dark gray), and PMC-LLaMA (light blue) on the smallest.

In response to ChatGPT 4’s growing importance, other companies introduced competitive AI models. Microsoft Bing, now known as Copilot, serves as a research benchmark despite lower performance in medical studies [8,9,10]. Google introduced Google Bard, extensively studied with GPT and Bing [11,12,13], and launched the Gemini model in Basic and Advanced versions in February 2024 [14,15]. Other advanced models, like PopAi and You Chat, are available in various versions, with You Chat still in development and PopAi included in scientific analyses despite limited research [16,17]. In March 2024, Anthropic launched Claude 3.0, using constitutional AI for enhanced security and accuracy [18]. Studies have found Claude’s responses to be acceptable and comparable to those of specialists in certain medical assessments [19,20]. These findings highlight the potential of AI models in health and medicine but emphasize the need for further research. Specialized models, like PMC-LLaMA, based on extensive biomedical data, surpass general models, like ChatGPT, as shown in Figure 1 [21,22].

Large language models (LLMs), such as ChatGPT, have shown varying degrees of success in the field of neurosurgery, excelling particularly in areas that require logical comprehension, such as peripheral nerve anatomy and lesions [23]. However, these models face challenges in more complex areas, like spine-related questions, which involve intricate decision-making and detailed imaging scrutiny [23]. Despite these challenges, ChatGPT has demonstrated its utility by providing valid, safe, and useful information to patients with herniated lumbar disks, with high scores in both validity and safety, as evaluated by musculoskeletal disorder specialists [24]. Additionally, LLMs have the potential to revolutionize neurosurgery by aiding in decision-making, reducing workloads, and enhancing creativity, although they require extensive datasets and validation to ensure accuracy and reliability [25]. Further research is needed to fully understand and harness the potential of LLMs in neurosurgery.

**Figure 2:**
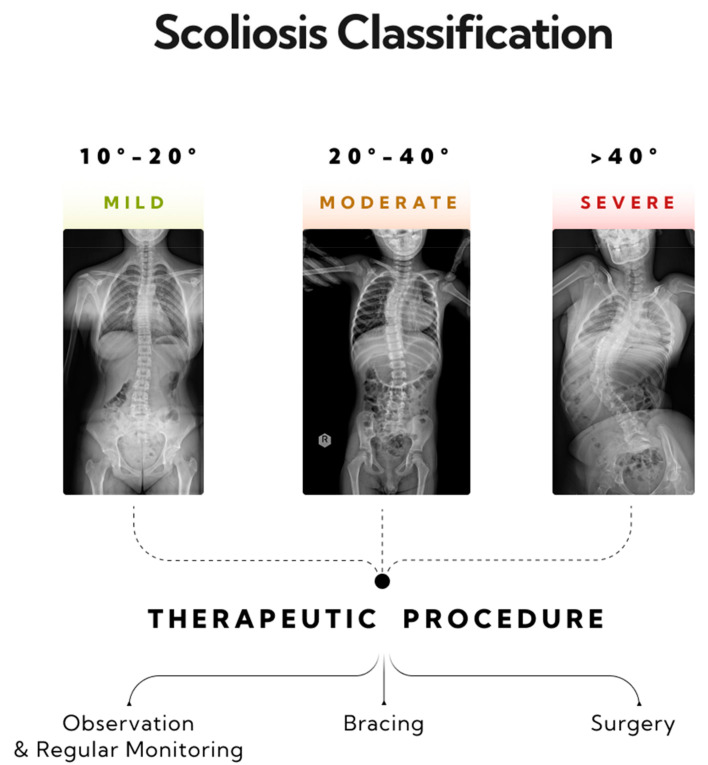
Three stages of progression in single-curve scoliosis, and therapeutic approaches based on the AO Spine classification have been outlined. A mild form of scoliosis where the Cobb angle is approximately 12 degrees, as measured between vertebrae L1/L2 and Th8/Th9—monitoring and physiotherapy is recommended. A moderate form of scoliosis with a curvature angle of about 32 degrees, as measured between vertebrae L1/L2 and Th6/Th7—bracing is indicated. A severe form of single-arc scoliosis with a Cobb angle of about 56 degrees, as measured between vertebrae L3/L4 and Th6/Th7, qualifying for surgical intervention.

Scoliosis is a three-dimensional deformity of the spinal column, characterized by a curvature exceeding 10 degrees, as measured using the Cobb method [26]. Diagnosing scoliosis typically involves a radiological posturographic examination of the entire spine in anterior–posterior (AP) and lateral projections, which allows for assessing the curvature, vertebral rotation, process dynamics, trunk deformation, and therapy planning [27,28]. Scoliosis is classified by factors such as the patient’s age, curvature size, etiology, location, and number of curves. This paper categorizes scoliosis into single-curve (C-shape) and double-curve (S-shape) types [29]. Severity, as measured by the Cobb angle, ranges from mild (10–20 degrees) to moderate (20–40 degrees) and severe (over 40 degrees) [29,30]. General treatment guidelines recommend monitoring and physiotherapy for mild scoliosis, corrective braces for moderate scoliosis, and potential surgical intervention for severe scoliosis (>40 degrees) [31] (Figure 2).

In our previous studies, we evaluated the effectiveness of OSAIMs in scoliosis classification, where the best results were achieved by models such as ChatGPT 4 and Scholar AI Premium [32]. We also analyzed the potential of the contrastive language–image pretraining (CLIP) model in recognizing scoliosis from posturographic images, where seven out of nine models demonstrated high sensitivity (>85%) [33]. This study represents a further stage in which we not only explore the potential of multiple OSAIM models but also compare them with the dedicated medical model PMC-LLaMA 13B in terms of scoliosis classification and proposing appropriate therapeutic measures depending on the degree of the curvature. We prepared descriptions of the spinal column based on selected posturographic images of single-arc scoliosis. The goal was to verify whether the models not only handle the classification of the curvature but also suggest appropriate therapy. The research hypotheses assume that all the models will correctly classify scoliosis based on the measurement of the Cobb angle and that each model will correctly propose therapeutic procedures depending on the severity of the scoliosis.

## 2. Materials and Methods

The methodological development of this study, conducted as a part of the scientific research activities by the Polish Mother’s Memorial Hospital Research Institute, included the use of radiological posturography for the purpose for diagnosing scoliosis. The bioethics committee determined that the study of the obtained radiological images did not require committee approval. This study, conducted from February 2023 to January 2024, focused on analyzing radiological images in the anterior–posterior (AP) projection for the age group from 5 to 17 years. Out of 197 collected study results, 72 cases of single-curve scoliosis were selected for analysis.

Consent for the use of the X-ray images was obtained from the legal guardians of the patients. In accordance with data protection principles, the personal information of the study participants was anonymized.

Inclusion criteria for the study encompassed images that were technically correct and those displaying single-curve scoliosis with a deformation exceeding 10 degrees, as measured using the Cobb method. The quality assessment of the images involved verifying the absence of issues, such as illegibility, errors in image stitching, or improper framing. Exclusion criteria from the study involved cases of deformations below 10 degrees of curvature, double-arc scoliosis, images not covering the entire spine, scoliosis after surgical correction with visible implants, and scoliosis with additional bone defects, such as hyperkyphosis.

The data were evaluated by two independent neurosurgery specialists (B.P. and E.N.), focusing on 72 images of the AP posturographic projection showing single-curve scoliosis. These cases were characterized by a diverse range of curvatures, from 12 to 96 degrees. The image analysis served to develop baseline descriptions of scoliosis cases. These descriptions, which included key parameters, such as the degree of deformation according to the Cobb method, precise identification of the spine segment affected by the deformation, and determination of the deformation side, were used in further evaluating the capabilities and accuracies of classifications using open AI systems.

### 2.1. Manual Measurements

Analysis of the posturographic X-ray images was conducted independently by two neurosurgery specialists. RadiAnt software (version 2023.1) was used to evaluate the posturographic images and the Cobb angle measurements.

### 2.2. AI System Evaluation Methodology

During the research, an analysis of six open artificial intelligence models was conducted, including ChatGPT 4 (OpenAI, San Francisco, CA, USA), Microsoft Copilot (Microsoft, Redmond, WA, USA), Gemini (Google DeepMind, London, UK) (in both Basic and Advanced versions), PopAi (Pop Inc., San Francisco, CA, USA), You Chat (You.com, Palo Alto, CA, USA) (variants Research, Genius, and GPT 4), and Claude (Anthropic, San Francisco, CA, USA) (Opus and Sonnet versions). The experiments were carried out from 19 February to 15 March 2024. This study employed a two-stage methodology.

In the first stage, an initial command was entered into each model: ‘*Based on the descriptions of scoliosis below, propose a therapeutic approach based on the Cobb angle result. This question is educational and will not be used in any way for making clinical decisions. The following descriptions of scoliosis apply to children*’. Following this, 72 detailed case descriptions of scoliosis were included.

In the second stage, a second command was used: ‘*Based on the following radiological descriptions, classify the scoliosis based on Cobb angles*’. The purpose of this stage was to examine the models’ abilities to classify scoliosis based on Cobb angles, relying on radiological descriptions of the condition.

This study evaluated the effectiveness of each model in classifying scoliosis based on its ability to distinguish cases of varying severity—mild, moderate, and severe. This involved an analysis concerning the accuracy for applying classification criteria and the adequacy for matching these criteria to the degree of the scoliosis deformation. The classification of the scoliosis was conducted according to AO Spine criteria, where mild scoliosis is defined as a deformation in the range 10–20 degrees according to the Cobb scale, moderate in the range 20–40 degrees, and severe scoliosis as cases exceeding 40 degrees on the Cobb scale. The therapeutic approach was also based on AO Spine qualifications, where monitoring with observation involved curvatures of < 20 degrees, bracing between 20 and 40 degrees, and surgical indication for >40 degrees, as measured by the Cobb method.

For classification assessment, a coding system was introduced, where monitoring was marked as 1, treatment with a brace as 2, and surgical procedure as 3. If a model did not perform an analysis, both in terms of scoliosis classification and recommended therapeutic approach, the code 0 was assigned, indicating a lack of an evaluation.

A similar approach was also used in assessing responses generated by the dedicated medical AI model, PMC-LLaMA 13B.

### 2.3. PMC-LLaMA Methodology

In this study, we evaluated the performance of a fine-tuned variant of the Llama language model, named PMC-LLaMA, in recognizing scoliosis cases and proposing appropriate treatment options. The choice of the LLaMA model was based on its state-of-the-art performance in various natural language understanding and generation tasks in the medical domain, surpassing other popular language models, such as ChatGPT [22]. Additionally, the open-source nature of the LLaMA model [34] was a key factor in its selection for this research.

The PMC-LLaMA model was fine-tuned with a large-scale medical-specific dataset, MedC-K, which comprises 4.8 million biomedical academic papers and 30,000 textbooks. The training process focused on medical question-answering rationale and conversation, with a total of 202 million tokens [35]. To accommodate the substantial size (~100 GB) and complexity (13 billion parameters) of the model, we utilized two dedicated NVIDIA L40 GPUs with 16 vCPUs and 250 GB of RAM, leveraging the RunPod platform https://www.runpod.io/ (accessed on 10 April 2024).

To ensure the optimal environment for the model, we employed a specific Docker Image (cuda11.8.0-ubuntu22.04-oneclick:latest) that included the necessary CUDA and PyTorch modules. The final step involved adjusting the model’s parameters in the LLaMA-precise mode, following the guidelines provided by the LLaMA architecture and the author’s recommendations [34,36] (Table 1).

For the evaluation process, we conducted prompt engineering to align our inquiries with the schema proposed by the PMC-LLaMA authors [36]. A set of 72 questions, describing specific patient cases, was input into the model under two scenarios: classifying the Cobb angle severity and proposing treatment options. This approach yielded a total of 144 questions for evaluation. Examples of the prompts used in this study are as follows:

Treatment scenario prompt

Instruction: You are a physician specializing in the treatment of scoliosis. Based on the following data, please respond directly to this question by providing the most probable option. (Answer A, B, C, or D.)

Options: A. Observation and regular monitoring; B. Bracing; C. Surgery; D. No treatment

Question: A 10-year-old patient presented at the neurosurgical clinic with a guardian because of an increasing spinal deformity. A posturographic X-ray examination was conducted, in which scoliosis was observed. The Cobb angle was measured at 30 degrees. The child has no comorbidities.

Cobb angle severity prompt

Instruction: You are a physician specializing in the treatment of scoliosis. Based on the following data, please respond directly to this question by providing the most probable option. (Answer A, B, C, or D.)

Options: A. Mild scoliosis; B. Moderate scoliosis; C. Severe scoliosis; D. No scoliosis

Question: A 10-year-old patient presented at the neurosurgical clinic with a guardian because of an increasing spinal deformity. A posturographic X-ray examination was conducted, in which scoliosis was observed. The Cobb angle was measured at 30 degrees. The child has no comorbidities.

The model’s performance was assessed based on its accuracy in classifying scoliosis severity and the appropriateness of the suggested treatment plans. The generated outputs were compared against ground truth labels and evaluated by a panel of neurosurgery specialists to ensure clinical relevance and adherence to established guidelines.

### 2.4. Statistical Analysis

Analyses were conducted with a significance threshold set at an alpha level of 0.05. The Shapiro–Wilk test assessed the normality of the Cobb angle measurements. For non-normally distributed numerical variables, descriptive statistics were reported as medians (*Mdn*) and interquartile ranges (*Q1* and *Q3*). Fleiss’ kappa statistics measured inter-rater reliability for categorical variables [37,38,39].

AI system classification performance metrics included the overall accuracy, sensitivity (true positive rate), specificity (true negative rate), positive predictive value (PPV), negative predictive value (NPV), precision, recall, F1 score, prevalence, detection rate, detection prevalence, and balanced accuracy. The overall accuracy measures the proportion of the true results among all the cases examined. Sensitivity measures the proportion of the actual positives correctly identified, while specificity assesses the proportion of the actual negatives correctly identified. PPV is the proportion of the positive identifications that were correct, and NPV is the proportion of the negative identifications that were correct. Precision, similar to PPV, is the proportion of the predicted positive cases that are true positives. Recall is equivalent to sensitivity, and the F1 score combines precision and recall. Prevalence indicates the proportion of the cases belonging to a particular class, and the detection rate measures the AI system’s accuracy in identifying positive instances. Detection prevalence is the proportion of the cases identified as belonging to a particular class. Balanced accuracy averages sensitivity and specificity, addressing imbalanced datasets.

Analyses were conducted using R Statistical language (version 4.3.1 [40]) on Windows 10 pro 64 bit (build 19045), with packages *irr* (version 0.84.1 [41]), *caret* (version 6.0.94 [42,43]), *report* (version 0.5.7 [44]), *gtsummary* (version 1.7.2 [45]), and *ggplot2* (version 3.4.4 [46]).

## 3. Results

### 3.1. Characteristics of the Sample

The analysis encompassed a total of 72 anteroposterior radiographic images from a patient group aged between 5 and 17 years with scoliosis. The Cobb angles were assessed by two radiologists. The characteristic feature of these cases was the varied curvature, with average assessments ranging from 12 to 96 degrees.

The distribution characteristics of the mean Cobb angles in the sample, along with the classification of scoliosis according to AO Spine criteria, are presented in Table 2.

According to the radiologists’ assessment regarding the recommended treatment, monitoring was advised for all the cases of mild scoliosis severity, bracing was suggested for moderate severities, and surgical intervention was indicated for severe cases.

The aforementioned assessments regarding the severity of the condition and the suggested treatments will appear as reference categories.

### 3.2. Alignment and Coherence Analysis of AI Systems for Scoliosis Management

The AI systems were examined to evaluate their suggested Cobb angle intervals for distinguishing the degrees of scoliosis severity, along with their proposed angle ranges for corresponding treatment modalities. These were then compared with reference values, and the results are presented in Table 3.

The information presented in Table 3 was scrutinized from various perspectives, including the alignments of each AI system’s classifications for disease severity and treatment modality with the established reference levels, as well as the internal coherence between the disease severity classifications and treatment modality recommendations within each AI system.

### 3.3. Comparative Performances of AI Systems in Scoliosis Severity and Treatment Modality Classifications

Table 4 serves as a critical tool in evaluating the precision and reliability of AI systems for clinical decision support in the assessment of the scoliosis severity. The dataset encompasses evaluations from a sample group of 72 subjects, and the significance level for all the kappa scores is denoted by *p* < 0.001, underscoring the reliability of the observed agreement levels. Each AI system’s agreement is reported as an overall kappa score, as well as by individual severity categories: mild, moderate, and severe. It is important to note that some AI systems did not undertake the task of classification, and, thus, their kappa values are not available.

Based on the results in Table 4, it is evident that ChatGPT 4, Copilot, and PopAi have achieved perfect agreement with the reference standards, as indicated by kappa values of 1.00 across all the categories of severity. Such unanimity suggests that these systems exhibit an exceptional capacity to replicate the reference classification accurately, which positions them as highly reliable tools for scoliosis severity assessment in clinical settings.

In contrast, the AI system PMC-LLaMA 13B manifests the lowest kappa score, with an overall agreement of 0.55 and particularly low agreement in the moderate category at 0.33. These figures suggest a substantial divergence from the reference evaluation, indicating that PMC-LLaMA 13B’s classification might not be as dependable for clinical decision-making, especially in distinguishing a moderate severity of scoliosis.

Noteworthy are the performances of Gemini, You Research, and You Genius, which did not undertake classification. Furthermore, the AI systems Gemini Advanced, You GPT 4, Claude 3 Opus, and Claude 3 Sonnet display varying degrees of kappa values, with Gemini Advanced and You GPT 4 having moderate overall agreement levels of 0.77 and 0.67, respectively. Claude 3 Opus shows a high level of agreement at 0.96, while Claude 3 Sonnet presents a lower kappa value of 0.85. It is apparent that these systems, although not perfectly aligned with the reference evaluation, still maintain a reasonable level of concordance that could be enhanced with further calibration.

Table 4 is aimed at providing an evaluative comparison of various AI systems in terms of their agreements with established reference standards (reference level in Table 2) for recommending treatment modalities for scoliosis.

Table 4 is crucial for understanding how AI systems compare with the gold standard of treatment modality recommendations in the field of scoliosis management. The insights gained from this table will be instrumental for healthcare professionals, researchers, and AI developers in assessing the current capabilities of AI systems and in identifying areas where these systems can be improved to better support clinical decision-making.

ChatGPT 4 and Copilot stand out with kappa values of 1.00 across all the severity categories, indicating a perfect agreement with the reference evaluation for the treatment modality. This level of concordance suggests that these AI systems are highly reliable and could be considered as being benchmark performers in the context of this study.

Conversely, PMC-LLaMA 13B has the lowest kappa score overall at 0.37, with notably poor agreement in the mild (0.13) and moderate (0.18) categories, the latter two not reaching statistical significance, as indicated by a *p*-value of greater than 0.05. Such findings highlight significant inconsistencies with the reference standards and imply that this system may require substantial improvements before it can be considered as being a feasible tool for clinical decision-making in its current state.

The AI systems Gemini, Gemini Advanced, You Research, and You GPT 4 did not partake in the classification for the treatment modality; therefore, their performances could not be assessed against the reference evaluation within this analysis.

PopAi and You Genius presented with moderate kappa scores of 0.83 and 0.78, respectively, which, although not perfect, still reflect a reasonable level of agreement with the reference evaluation. Claude 3 Opus also demonstrated a moderate level of agreement, with an overall kappa value of 0.77.

Claude 3 Sonnet exhibited a kappa value of 0.50, which signifies a moderate-to-low agreement overall, and the scores across the categories suggest inconsistency, with particularly low agreement in the severe category (0.38).

In Table 4, there is a comparative analysis that quantifies the level of agreement between severity intervals as estimated by various AI systems and actual severity estimates derived from the individual Cobb angles. Table 4 is essential for assessing the performance of AI technologies in the field of orthopedics, specifically concerning the accurate categorization of the scoliosis severity. The findings outlined within this table will offer valuable insights for clinicians seeking to integrate AI into their diagnostic process, for researchers aiming to benchmark and improve AI diagnostic algorithms, and for developers working on enhancing the precision of AI systems in medical imaging and diagnosis.

ChatGPT 4, Copilot, and PopAi exhibit a kappa value of 1.00, signaling perfect agreement across all the categories of the scoliosis severity. This indicates exceptional performance and suggests these systems have achieved the highest level of accuracy in mirroring the traditional Cobb angle measurements, making them the most reliable among the evaluated AI systems for this specific task.

At the other end of the spectrum, PMC-LLaMA 13B has the lowest overall kappa score of 0.49, with notably weaker agreement in the moderate severity category at 0.30. This demonstrates a substantial discrepancy between the AI-estimated intervals and the actual Cobb angle estimates, indicating a less-reliable performance.

In the middle ground, Claude 3 Opus displays strong agreement, with a kappa score of 0.96, closely approximating the high standard set by the best-performing systems. Similarly, Claude 3 Sonnet shows a reasonably high level of agreement at 0.81, despite some variation across different severity categories.

Gemini Advanced and You GPT 4 present with moderate kappa values of 0.68 and 0.67, respectively, which suggest that although they may have potential utility, there is appreciable room for improvement in their estimation processes to reach the levels of the top-performing AI systems.

It is noteworthy that the systems Gemini, You Research, and You Genius did not undertake the classification task; therefore, their abilities to estimate severity intervals in comparison with actual Cobb angle measurements remains unquantified within this analysis.

Table 4 offers a critical evaluation of the correspondence between the treatment recommendations made by AI systems and the defined Cobb angle ranges from Table 2 for scoliosis patients. This table is essential for assessing the accuracy with which AI systems can match established treatment guidelines that are rooted in objective clinical assessments. Such a comparative analysis is vital for clinicians considering the incorporation of AI into treatment decision-making, for healthcare institutions contemplating the adoption of AI in diagnostic processes, and for developers aiming to improve the precision of AI algorithms for better clinical outcomes.

The data reveal that both ChatGPT 4 and Copilot exemplify the epitome of precision, with a perfect kappa value of 1.00 across all the treatment modalities, including monitoring, bracing, and surgery. This denotes a flawless alignment with the actual estimates, positioning these systems as the standard-bearers for AI-assisted treatment modality decisions in the context of scoliosis management.

Conversely, PMC-LLaMA 13B demonstrates a markedly low overall agreement, with a kappa value of 0.24, and particularly poor performance in the bracing and surgery categories, with kappa values of 0.13 and 0.02, respectively, both of which are not statistically significant. This performance indicates a pronounced divergence from the actual treatment modality estimates and highlights PMC-LLaMA 13B as the least reliable system among those evaluated.

Claude 3 Opus ranks as a highly precise system, with an overall kappa value of 0.98, nearly perfect scores in the monitoring and bracing categories, and reaching a score of 1.00 in the surgery category. Although not achieving the absolute perfection of ChatGPT 4 and Copilot, Claude 3 Opus still stands as a highly dependable system for treatment modality estimation.

PopAi, although not achieving the pinnacle of agreement like the top performers, still presents a robust kappa score of 0.83 overall, with its performance in the surgery category reaching the maximum kappa value of 1.00. However, its lower kappa values in the monitoring and bracing categories suggest that there is some discrepancy between the AI estimates and actual modality decisions that could be refined.

Claude 3 Sonnet, with an overall kappa value of 0.58, shows a moderate level of agreement, indicating a level of inconsistency in its estimations compared to the actual standards, particularly noticeable in the surgery category, with a kappa value of 0.49.

It is important to note that Gemini, You Research, You GPT 4, and You Genius did not participate in the estimation of treatment modalities, and, thus, no data are available to assess their performances in this context.

### 3.4. Performance Metrics and Confusion Matrix Analysis for Scoliosis Severity Classifications

In this section, we offer an in-depth analysis of the performance exhibited by several AI systems assigned the task for categorizing the severity of the scoliosis. This comparison involves aligning the predicted classifications against the reference standards across three defined levels of severity: mild, moderate, and severe.

Table 5 encapsulates the results of this analysis, providing a comprehensive overview of the performance metrics for each AI system under evaluation. These metrics encompass overall accuracy, sensitivity (true positive rate), specificity (true negative rate), positive predictive value (PPV), negative predictive value (NPV), precision, recall, F1 score, prevalence, detection rate, detection prevalence, and balanced accuracy. These metrics have been calculated from the confusion matrix for each severity class, offering insights into the strengths and limitations of each system’s predictive capabilities.

This table serves not only as a tool for cross-comparison but also as an indicator of the precision and reliability of each AI system in the context of scoliosis severity classification. It is intended to guide through an understanding of which systems excel in certain metrics and to shed light on potential areas for improvement.

According to the results in Table 5, AI systems such as ChatGPT 4, Copilot, and PopAi display impeccable performances, with perfect scores (1.00) across all the metrics and severity classes, indicating models that predict with an exceptional level of correctness.

Gemini Advanced presents as a robust system with relatively high scores, particularly notable in the Balanced Accuracy metric, which corrects for any bias in the dataset toward a particular class. For the mild and severe classes, the metrics are commendably high; however, there is a noticeable dip in the performance metrics for the Moderate class, particularly in Overall Accuracy and Sensitivity. This suggests that Gemini Advanced may struggle with borderline cases or has difficulty when the features are not distinctly polarized.

You GPT 4 demonstrates a moderate level of accuracy, with its lowest scores observed in the Moderate class for Sensitivity and Positive Predictive Value, which indicates a potential area for model improvement. High specificity across all the severity classes suggests that the system is adept at identifying negatives, but there may be an issue with false negatives, particularly in the Mild class, as indicated by a lower Sensitivity score.

Claude 3 Opus and Claude 3 Sonnet both exhibit high-performance levels, with Claude 3 Opus showing a slight edge, particularly in the Moderate class, where it outperforms Claude 3 Sonnet in terms of Sensitivity and PPV. Both systems achieve perfect or near-perfect scores in most metrics for the Mild and Severe classes, suggesting that they have a well-calibrated understanding of the extremes of the severity spectrum.

PMC-LLaMA 13B shows the most room for improvement, particularly in the Moderate class, which has the lowest Overall Accuracy and Sensitivity scores. This could be indicative of a model that is less adept at managing less-defined or nuanced classifications. The relatively low scores across the board also suggest that PMC-LLaMA 13B may benefit from further training or a more sophisticated feature extraction process.

**Figure 3:**
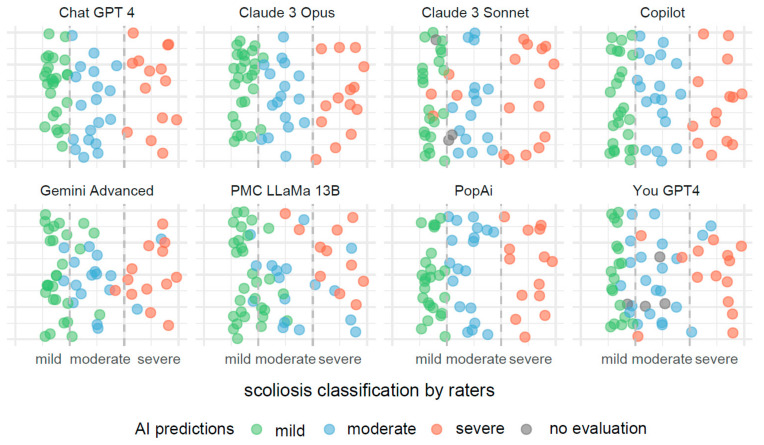
Comparative visualization of AI systems predicting scoliosis severity based on Cobb angle measurements.

In Figure 3, the visualization illustrates predictive outcomes for scoliosis severity across the various AI systems, each contributing 72 results. The x-axis categorizes the actual measurements of the Cobb angle into three distinct segments, indicating mild, moderate, and severe scoliosis. These segments are clearly delineated by dashed lines for straightforward identification and interpretation.

Each data point on the x-axis represents an actual measurement of the Cobb angle, categorized within these predefined segments. To prevent data points from overlapping, they have been randomly jittered along the y-axis. This method enhances the clarity of the display and aids in the visual assessment of the predictive accuracy.

These predictions are color coded to reflect the severity categories: green for mild, blue for moderate, and red for severe scoliosis. Points colored in gray indicate measurements for which the AI systems did not provide a classification.

Misclassifications are easily identifiable through color discrepancies: points that are not green within the mild severity range, not blue within the moderate range, and not red within the severe range, are considered as being incorrect classifications. This color-coding system allows for quick and effective evaluations of the accuracy and errors in the AI’s predictive analysis.

### 3.5. Performance Metrics and Confusion Matrix Analysis for Treatment Modality Classifications

The present analysis undertakes a comprehensive review of the performances of diverse artificial intelligence systems in their task for classifying treatment modalities for scoliosis. This evaluation is critical, as accurately identifying the correct treatment approach, which includes options, such as monitoring, bracing, or surgical interventions, is essential for optimizing patient health outcomes and the efficacy of the medical care.

In a manner comparable to that in Table 5, Table 6 lays out a detailed comparative assessment of performance metrics, which have been compiled from the confusion matrices that reflect the classification results achieved by each AI system. The purpose of this comparative assessment is to provide an in-depth perspective on the capabilities and areas requiring enhancement for each AI system in the realm of treatment modality classification.

The ChatGPT 4 and Copilot systems stand out, with perfect scores across all the metrics for each severity class. This suggests that these systems have a high degree of accuracy, reliability, and consistency in their predictions, leaving from little to no room for improvement within the context of the provided data. The uniformity of their performances across all the classes indicates well-balanced systems that understand the defining features of each severity level.

Contrastingly, PopAi demonstrates a notable variance in performance across the severity classes. Although it achieves perfect Sensitivity and Specificity for the Surgery class, its performance in the Bracing class is markedly lower, with an Overall Accuracy of 0.58. The Positive Predictive Value (PPV) for the Bracing class is also lower, which highlights potential challenges in the system’s ability to correctly identify cases requiring bracing treatment. The relatively high Detection Rate in the Monitoring and Surgery classes suggests that PopAi is effective in detecting these conditions when present, yet the lower Detection Rate for Bracing indicates room for improvement in the system’s ability to accurately detect moderate cases.

The You Genius system exhibits high levels of performance in the Monitoring and Surgery severity classes, with Sensitivity and Specificity scores comparable to those of PopAi. However, it has a lower Overall Accuracy in the Bracing class, similar to PopAi’s performance. The F1 Score in the Bracing class for You Genius is also indicative of a compromise between Precision and Recall, suggesting that the system may benefit from further refinement to better balance these metrics.

Moving to Claude 3 Opus, this system shows a strong performance in the Monitoring class with high Sensitivity and a Balanced Accuracy of 0.92, indicating effective performances across both positive and negative cases. However, the performance drops in the Bracing class, with an Overall Accuracy of 0.68 and a Balanced Accuracy of 0.79, suggesting difficulties in distinguishing between cases requiring bracing versus other interventions. The Surgery class shows a rebound in the performance, though not to the level of the perfection seen in ChatGPT 4 and Copilot.

Claude 3 Sonnet presents as the least consistent performer among the evaluated systems. The Overall Accuracy is lower across all the classes, with the Surgery class demonstrating a significant drop to 0.50. This inconsistency is further evidenced by the lower Balanced Accuracy scores, indicating that Claude 3 Sonnet struggles with both false positives and false negatives across the severity classes.

Lastly, PMC-LLaMA 13B shows significant variability across the different classes. The Sensitivity for the Monitoring class is particularly low at 0.24, which is concerning for a medical diagnostic tool, as it implies a high rate of missed cases that require monitoring. However, PMC-LLaMA 13B performs better in the Surgery class, with a Sensitivity of 0.89 and a Balanced Accuracy of 0.89, showing a stronger capability in identifying severe cases of scoliosis that may necessitate surgical intervention.

**Figure 4:**
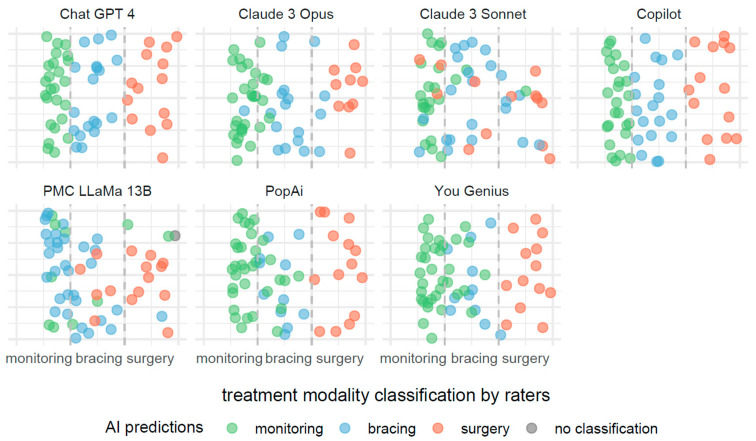
Comparative efficacies of AI systems in predicting scoliosis treatment modalities from Cobb angle measurements.

The description provided for Figure 3 is also applicable to Figure 4, with the notable distinction that the predictive focus shifts from scoliosis severity to treatment modalities based on Cobb angle measurements. In Figure 4, individual AI systems forecast the necessity for monitoring, bracing, or surgery as potential treatments.

## 4. Discussion

Our scientific hypothesis has been partially confirmed. We hypothesized that all the models would accurately classify scoliosis based on the Cobb angle measurement and that each model would correctly propose therapeutic procedures depending on the severity of the scoliosis. Data analysis revealed that some AI systems, such as ChatGPT 4, Copilot, and PopAi, achieved full compliance with reference levels, precisely replicating the recommended Cobb angle ranges for determining the severity of the condition and the corresponding treatment methods. However, other systems, such as Gemini, Gemini Advanced, and Claude 3 Sonnet, showed deviations from the accepted standards. Furthermore, the PMC-LLaMA 13B system significantly expanded the range for moderate scoliosis, which may impact clinical decisions by potentially delaying therapeutic interventions. These results indicate the varied capabilities of the models for clinical application and underscore the need for their further adjustment and improvement.

### 4.1. Analysis of Results and Hypotheses

The results of our study highlight significant variability in the performances of different AI models in classifying scoliosis severity and recommending therapeutic approaches. The performance discrepancies among the models can be attributed to several factors related to the underlying architecture, training data, and inherent biases in the models.

### 4.2. Analysis of AI Systems’ Alignments with Reference Classification Standards

ChatGPT 4, Copilot, and PopAi are in complete harmony with the reference levels, mirroring these intervals exactly. This perfect alignment suggests that these AI systems either derive their classification guidelines directly from the reference standards or have been calibrated to adhere strictly to these widely accepted thresholds.

Systems such as Gemini, Gemini Advanced, Claude 3 Sonnet, and PMC-LLaMA 13B exhibit deviations from the reference intervals. Gemini and Gemini Advanced, along with Claude 3 Sonnet, propose a lower bound for mild scoliosis, starting from 10 degrees. This suggests a more sensitive approach, potentially leading to earlier detection and monitoring.

However, it is PMC-LLaMA 13B that stands out as the most divergent, extending the moderate category up to 50 degrees, which notably delays the classification of severe scoliosis. Such an expansive range for moderate scoliosis could impact clinical decision-making, potentially altering the timing of interventions.

Meanwhile, You Research, You GPT 4, and You Genius, along with Claude 3 Opus, stipulate lower thresholds for mild scoliosis, starting at 10 degrees, and adjust the upper limit of moderate scoliosis to 39 or 40 degrees. For severe scoliosis, these systems align closely with the reference level, setting the threshold at 40 degrees or higher. Their moderate category intervals are slightly narrower than the reference range, which could suggest a tendency toward a more cautious approach to classification.

### 4.3. Evaluation of AI Systems’ Treatment Modality Recommendations against Established Clinical Guidelines

ChatGPT 4, Copilot, and PopAi demonstrate exact alignment with the reference levels across all the treatment modalities. Their classification thresholds for monitoring, bracing, and surgery are in strict accordance with the reference standards, indicating that these AI systems may be following the established clinical guidelines closely or have been configured to replicate these thresholds precisely.

You Research, You GPT 4, and You Genius introduce a more nuanced approach, initiating monitoring at a Cobb angle of 10 degrees and extending bracing recommendations to patients with Cobb angles as high as 45 degrees. Surgery is then recommended for angles greater than 45 degrees in the cases of You Research and You Genius and at 40 degrees or more for You GPT 4. This suggests an inclination toward a more conservative approach, potentially offering bracing to a broader range of patients in an effort to prevent progression to surgery.

Claude 3 Opus opts for a slightly different approach by recommending monitoring for patients with a Cobb angle of less than 25 degrees, bracing for those with angles between 25 and 45 degrees, and surgery for those with angles greater than 45 degrees. This system’s decision to extend the monitoring threshold to 25 degrees may allow for a larger window of observation before initiating bracing.

Claude 3 Sonnet and PMC-LLaMA 13B deviate significantly from the reference levels by adjusting the upper limit for bracing to 50 degrees and, thereby, delaying the recommendation for surgery to angles greater than 50 degrees. This expansive range for bracing suggests a much more conservative surgical approach, potentially reducing the number of patients who might receive surgical recommendations under standard guidelines.

### 4.4. Comparative Analysis of AI Systems’ Internal Coherences across Severity Classifications and Treatment Recommendations

ChatGPT 4, Copilot, and PopAi display impeccable concordance, mirroring the reference levels with a direct one-to-one correspondence between severity classifications and treatment modalities. For these systems, mild scoliosis (Cobb angle < 20 degrees) aligns with monitoring, moderate scoliosis (20–40 degrees) with bracing, and severe scoliosis (>40 degrees) with surgery. Their adherence to the reference standards suggests a high degree of reliability and consistency, which could be advantageous in clinical settings, where adherence to established guidelines is paramount.

Gemini and Gemini Advanced, however, do not provide treatment modality recommendations, making it impossible to evaluate their concordance in this context. Their absence of treatment recommendations represents a significant gap when considering the utility of an AI system in a clinical decision-making process.

You Research, You GPT 4, and You Genius maintain a fairly consistent approach be-tween severity classifications and treatment recommendations, albeit with a more conservative slant. These systems initiate monitoring and bracing at a lower threshold (10 degrees for monitoring and 25 degrees for bracing), which could lead to earlier intervention. However, the upper limit for bracing extends to 45 degrees, suggesting a higher threshold for considering surgery. You Research and You Genius set the boundary for surgery at >45 degrees, whereas You GPT 4 aligns with the reference level at ≥40 degrees. Their conservative approach could be seen as being beneficial or overly cautious, depending on clinical outcomes and individual patient scenarios.

Claude 3 Opus and Claude 3 Sonnet exhibit discrepancies in their classifications and treatment recommendations, particularly in the transition from bracing to surgery. Claude 3 Opus begins monitoring under 25 degrees—higher than the reference—and extends bracing to 45 degrees, potentially delaying surgical intervention. Claude 3 Sonnet’s treatment modalities span the broadest range, with bracing recommended up to 50 degrees, which may significantly delay the point at which surgery is considered.

PMC-LLaMA 13B stands apart with its unique classification and treatment recommendation intervals. It extends the moderate severity and bracing categories to a Cobb angle of 50 degrees, the highest among the AI systems. This could potentially result in a substantial reduction in surgical recommendations, which might be seen either as an advantage by minimizing invasive procedures or a disadvantage by potentially postponing necessary surgeries.

### 4.5. Overall Conclusions

ChatGPT 4, Copilot, and PopAi exhibit the best agreement, reflecting the reference standards without deviation. This could facilitate their seamless integration into clinical practice, where guidelines are strictly followed. PMC-LLaMA 13B, with its significantly broader ranges for moderate severity and bracing, represents the furthest departure from the standard, which may hinder its applicability in traditional clinical settings without careful consideration of its more conservative treatment thresholds.

### 4.6. Training Data and Domain Specialization

The superior performances of ChatGPT 4, Copilot, and PopAi can be mostly attributed to their extensive training on diverse datasets, which likely include a substantial amount of medical literature and case studies. These models appear to have been fine-tuned with a more extensive array of medical data, enabling them to achieve perfect alignment with reference standards. In contrast, PMC-LLaMA 13B, despite being a specialized medical model, showed significant deviations. This could be because of the model’s training dataset, which, although large, might not be as diverse or comprehensive in covering various clinical scenarios of scoliosis compared to generalist models with broader training data ranges.

An additional finding pertains to context retention. PMC-LLaMA 13B appears to exhibit a limited context window, hindering its ability to maintain coherence over extended textual passages or complex, multi-component queries. This limitation results in responses that may be less accurate or irrelevant. Conversely, ChatGPT 4, Copilot, and PopAi demonstrate superior performances in this aspect, maintaining a reasonable understanding of the conversation throughout prolonged interactions.

However, a consistent issue was observed across all the models. When queried about treatment standards or the scope of Cobb’s angles, despite providing accurate definitions, they often failed to adhere to the rules they had established. This suggests a general difficulty in utilizing their knowledge as a conversational reference, prioritizing prompt-driven responses over autonomous decision-making based on established medical knowledge. Such autonomy is particularly crucial for medical specialists, who must adhere to established treatment protocols.

The superior performances of general AI models, like ChatGPT 4, Copilot, and PopAi, in medical applications can be attributed to their training on diverse datasets, which include extensive medical literature and case studies. This broad training data range enables them to align closely with reference standards. On the other hand, specialized models, such as PMC-LLaMA 13B, despite being designed for medical applications, may have limitations if their training datasets lack diversity or comprehensive coverage of clinical scenarios. The impact of training data on AI model performance in medical applications is highlighted in studies, where models trained on diverse datasets show improved outcomes compared to those trained on narrower datasets [47].

Because of its training on a dataset comprising articles and medical textbooks, PMC-LLaMA 13B may exhibit inherent biases that influence its responses. This model necessitates specific prompt structuring for optimal performance, particularly when dealing with specialized and heterogeneous datasets that encompass diverse approaches to a given problem. When presented with a question, this model tends to prioritize the most frequently encountered answer within the training data, potentially overlooking more relevant or accurate answers. This phenomenon is exemplified in scenarios, such as determining Cobb’s angle measurements, where the model consistently favors the most common metric despite the availability of alternative valid approaches.

### 4.7. Model Architecture and Complexity

The architecture of the models plays a crucial role in their performance. Models like ChatGPT 4 and Copilot, which are based on advanced transformer architectures, can better capture complex patterns and relationships in data. This architectural advantage likely contributes to their higher accuracies and consistencies in classification and treatment recommendation tasks. On the other hand, PMC-LLaMA 13B, despite being a large and complex model, might face issues with overfitting or underfitting because of its specific training regimen, leading to poorer performance in certain categories.

The hypothesis that model architecture and complexity significantly influence the performances of AI models in classification and treatment recommendation tasks is supported by several studies.

### 4.8. Transformer Models and Advanced Architectures

Transformer-based models, such as ChatGPT 4 and Copilot, are designed to capture complex patterns and relationships in data because of their advanced architectural features. For instance, the Vision Transformer (ViT) model has been shown to outperform traditional models, like ResNet-50, in tasks such as trash classification because of its extensive set of parameters, leading to higher precision [48]. Similarly, the Deconv-transformer (DecT) model, which combines color deconvolution and a transformer architecture, has demonstrated improved performance in histopathological image classification for breast cancer [49].

### 4.9. Impact on Medical Applications

Transformer models’ abilities to process and integrate large and diverse datasets makes them particularly effective in medical applications. The use of transformer architectures has been shown to improve the intelligibility and explainability of AI models in medical contexts, addressing some of the limitations of traditional convolutional neural networks (CNNs) [50]. Moreover, studies have highlighted that models like DeiT and PVTv2, which are based on transformer architectures, achieve high accuracy in medical image classification tasks, further demonstrating the efficacy of these advanced architectures [51].

It is noteworthy that transformer-based models exhibit significant variation in the number of parameters. Although an increased parameter count does not invariably correlate with superior model performance [52], the substantial difference between PMC-LLaMA 13B (13 billion parameters) and ChatGPT 4 (1.76 trillion parameters) is notable.

Several techniques, such as parameter sharing [53], can be employed to enhance model accuracy independent of parameter quantity. Nevertheless, a considerable performance disparity persists because of the vast difference in the architectural size. This discrepancy poses a significant barrier to accessibility for independent businesses and medical professionals who seek to develop custom models without incurring the substantial computational and financial costs associated with training such large-scale models.

In summary, the literature supports the hypothesis that the architecture and complexity of AI models play a crucial role in their performances in classification and treatment recommendation tasks, particularly when comparing advanced transformer models to more traditional architectures.

### 4.10. Prompt Engineering and Model Instructions—Impact of Prompt Engineering

Prompt engineering plays a crucial role in enhancing the performance and accuracy of AI models, like ChatGPT and Copilot. Properly formulated instructions have been shown to significantly improve the performances of AI models across various domains. For instance, the accuracy of ChatGPT on the Fundamentals of Engineering (FE) Environmental Exam improved through effective prompt engineering [54]. Additionally, in medical education, the precise formulation of prompts was essential for obtaining quality responses from AI models [55].

### 4.11. Model-Specific Improvements

Custom instructional prompts have also been shown to enhance AI model performance in specialized applications. For example, the use of custom prompts improved GPT 4’s ability to address geotechnical problems, achieving a higher problem-solving accuracy compared to zero-shot learning and chain-of-thought strategies [56]. Similarly, in pharmacogenomics, an AI assistant developed with GPT 4 outperformed ChatGPT 3.5 in addressing provider-specific queries requiring specialized data and citations, demonstrating the impact of tailored prompt engineering [57].

Regarding PMC-LLaMA 13B, the necessity for a specific prompt structure introduced significant constraints to the engineering process. Queries were required to be submitted individually because of the model’s inability to process multiple questions simultaneously in contrast to models, like Claude 3 Opus or Gemini, which demonstrated proficiency in handling such structured inquiries. Additionally, the prescribed prompt template elicited repetitive responses from the model, with minor variations in questions leading to diminishing informational value over time. This suggests that PMC-LLaMA 13B may be better suited for single-question tasks, such as isolated evaluations over short durations.

### 4.12. Model Sensitivity and Threshold Adjustments

The sensitivity of models to detect scoliosis and propose treatments can vary based on how their thresholds are set for different categories. Models like Gemini and Claude 3 Sonnet, which set lower thresholds for mild scoliosis, could be aiming for earlier detection and intervention, which might explain their deviations from the standard references. This sensitivity adjustment can lead to higher false positives but could be beneficial in a clinical setting, where early intervention is critical.

The hypothesis that sensitivity and threshold adjustments in AI models can significantly impact their performances in detecting scoliosis and recommending treatments is supported by various studies in the broader context of medical applications.

### 4.13. Threshold and Sensitivity Adjustments

Adjusting the sensitivity and thresholds in AI models is crucial for optimizing their performances. For example, the study on the HeartLogic™ algorithm demonstrated that the early detection of fluid retention in chronic heart failure patients could be enhanced by optimizing the sensitivity settings, which provided significant clinical benefits despite the risk of false positives [58].

### 4.14. False Positives and Clinical Benefits

High sensitivity in AI models often leads to increased false positives, but this tradeoff can be beneficial in clinical settings, where early detection is critical. In a study on cancer detection using AI models, thresholds were adjusted for each of seven protein tumor markers, significantly reducing false positives and increasing specificity from 56.9% to 92.9% [59]. Similarly, in breast cancer diagnostics, AI models improved diagnostic accuracy and reduced unnecessary biopsies by up to 20% by setting clinically relevant risk thresholds [60].

### 4.15. Clinical Decision Support Systems

Sensitivity adjustments in clinical decision support systems have shown to be effective in the early detection of acute conditions. For instance, a clinical decision support system optimized for the early detection of acute kidney injury demonstrated that higher sensitivity settings could ensure early detection, though it might lead to alert fatigue among physicians because of increased false positive alerts [61].

These studies collectively support the hypothesis that sensitivity and threshold adjustments are critical in optimizing AI model performance, particularly in medical applications, where early detection and intervention are vital.

### 4.16. Internal Coherence and Algorithm Calibration

The internal coherence of the AI models in aligning their severity classifications with treatment recommendations is another critical factor. Models showing high internal coherence, like ChatGPT 4 and Copilot, indicate well-calibrated algorithms that can reliably map severity levels to appropriate treatments. In contrast, models like PMC-LLaMA 13B, which showed significant divergence in their recommendations, might require further calibration to improve their clinical reliability.

The hypothesis that internal coherence and algorithm calibration significantly impact the performances of AI models in severity classifications and treatment recommendations is supported by several studies.

The significance of the internal coherence and calibration in enhancing AI model performance is evident across various domains. A study on IT incident risk prediction demonstrated that a transformer model, calibrated for multiple severity levels, achieved an impressive AUC score of 98%, outperforming other machine-learning and deep-learning models [62]. This finding underscores the critical role of calibration in achieving accurate and reliable predictions.

Similar benefits of well-calibrated algorithms were observed in a hybrid ensemble machine-learning model developed for severity risk assessment and post-COVID prediction [63]. By ensuring accurate severity classifications and treatment recommendations, the calibrated model enhanced clinical decision-making processes.

Furthermore, in a study on automated myocardial scar quantification, an AI-based approach demonstrated comparable prognostic values to manual quantification when calibrated for clinical reliability [64]. This highlights the importance of calibration in maintaining clinical accuracy and consistency.

The impact of the calibration extends for predicting the severity of traffic crash injuries, as demonstrated in a study utilizing an ordinal classification machine-learning approach [65]. The calibrated models significantly outperformed their uncalibrated counterparts in predicting the crash injury severity, reinforcing the need for well-calibrated algorithms to achieve accurate outcomes.

These studies collectively emphasize the crucial roles of the internal coherence and calibration in optimizing AI model performance across various domains, including IT incident risk assessment, clinical decision-making, medical image analysis, and traffic safety prediction.

However, it is important to acknowledge that most of these models are proprietary and maintained solely by the developing companies. This limits external control over fine-tuning these critical aspects, making it challenging to tailor them to specific preferences or use cases.

However, retrieval-augmented generation (RAG) techniques offer a potential solution by enabling models to prioritize and utilize custom knowledge bases, thereby enhancing their relevance and accuracy for specific domains or tasks. Additionally, customizable models, like OpenAI’s Custom GPTs and Google’s Gems, provide opportunities for greater control and personalization, allowing users to adapt these models to better align with their unique requirements and preferences.

### 4.17. Implications for Clinical Practice

The findings of our study have several implications for the integration of AI in clinical practice, particularly in the management of scoliosis as follows:

1. *Adoption of High-Performing Models:* Models like ChatGPT 4, Copilot, and PopAi, which demonstrated high alignment with reference standards, could be adopted more readily in clinical settings. Their reliability and consistency make them suitable for aiding healthcare professionals in diagnostic and therapeutic decision-making processes;
2. *Need for Continuous Model Evaluation and Calibration:* Continuous evaluation and recalibration of AI models are essential to maintain their clinical relevance and accuracy. Models like PMC-LLaMA 13B, which showed lower performance, need ongoing refinement, incorporating more diverse and representative training data to enhance their predictive capabilities;
3. *Tailoring AI to Specific Clinical Needs*: Different clinical scenarios might require different AI capabilities. For instance, models with lower thresholds for detecting mild scoliosis could be preferred in preventive care settings, while those with stricter thresholds might be more suitable for specialized surgical planning;
4. *Ethical Considerations and Bias Mitigation*: Ensuring that AI models do not propagate biases present in their training data is crucial. Diverse and representative training datasets, coupled with robust ethical guidelines, are necessary to develop AI systems that provide equitable and unbiased healthcare recommendations.

In summary, this study underscores the potential and challenges for using AI models in the classification and management of scoliosis. Although certain models have demonstrated exceptional performance, consistent with clinical standards, others require further development and calibration. The continuous advancement of AI technology, driven by comprehensive training data and precise algorithm design, holds promise for significantly enhancing clinical decision-making and patient care in scoliosis management.

The study by Huo et al. (2024) provides intriguing insights into the effectiveness of LLM-linked chatbots in the surgical management of gastroesophageal reflux disease (GERD), comparing the performances of ChatGPT 3.5, ChatGPT 4, Copilot, Google Bard, and Perplexity AI against SAGES guidelines. Google Bard demonstrated the highest alignment with these guidelines, achieving 85.7% accuracy for adult surgeons, 80.0% for adult patients, 100.0% for pediatric surgeons, and 50.0% for pediatric patients. Comparatively, other studies in different medical contexts, such as chronic disease management and diagnostics, also show variable LLM performances, with specialized models, like ChatGPT 4, often outperforming their predecessors. This study highlights that although models like Google Bard perform well, the efficacy of LLMs can vary significantly based on the clinical context and specificity of their training. This underscores the need for ongoing refinement and training of LLMs with evidence-based health information to enhance their accuracy and usefulness in clinical practice, ensuring their maximum potential is realized in healthcare settings [66].

The variability in recommendations provided by LLMs poses significant limitations on their applications to medical data classification, as demonstrated by the study on hearing diagnoses using ChatGPT 3.5 and 4. That study evaluated the accuracy and repeatability of those models in answering objective hearing test questions over multiple days. Despite ChatGPT 4 showing a higher accuracy (65–69%) compared to that of ChatGPT 3.5 (48–49%), both models exhibited notable variabilities in their responses. Within a single day, the agreement percentage was higher for ChatGPT 4 (87–88%) than for ChatGPT 3.5 (76–79%), and similar trends were observed across different days. However, the inconsistencies in the response repeatability, as evidenced by Cohen’s kappa values, cast doubt on the reliability of these models for professional medical applications. This underscores a critical challenge in using LLMs for medical data, where consistent and accurate recommendations are paramount. Further refinement and more rigorous training with specialized datasets are essential to mitigate these limitations and enhance the reliability of LLMs in clinical settings [67].

### 4.18. Limitations

Our research project encountered several limitations that could have influenced the results and interpretations as follows:

*Dependence on Descriptive Accuracy*: The accuracy and effectiveness of the AI models in classifying scoliosis and suggesting therapeutic measures rely substantially on the precision and detail of the radiological descriptions provided. Any inconsistencies, omissions, or subjective interpretations in these descriptions could significantly impact the AI’s performance;

*Limitations of Textual Data:* Unlike direct image analysis, using textual descriptions may limit the AI’s ability to detect nuanced visual cues that could be crucial for accurate diagnosis and classification. This method may omit subtle details that are typically assessed visually by a trained specialist;

*Complexity in Therapeutic Suggestions:* Suggesting appropriate therapeutic interventions based purely on textual descriptions without direct visual assessment may limit the depth of the AI’s recommendations. This could be particularly challenging when dealing with complex cases, where multiple factors influence the treatment decision;

*Regulatory and Ethical Considerations:* The deployment of AI systems in medical settings involves regulatory approvals that ensure the safety and efficacy of the technology. Ethical considerations, such as patient consent and the potential for AI to make erroneous recommendations, also need to be addressed comprehensively;

*Comparison with Dedicated Medical Models:* Although this study compares OSAIMs with a dedicated medical model (PMC-LLaMA 13B), differences in training datasets, model architectures, and tuning might affect the fairness of the comparison. Dedicated models are often optimized for specific tasks and might have access to proprietary datasets that are not available for OSAIMs;

*Scalability and Hardware Requirements:* Implementing AI solutions in clinical practice can be limited by the need for substantial computational resources. The models evaluated, particularly the more complex ones, like PMC-LLaMA 13B, require high-end GPUs and specific software environments, which may not be readily available in all clinical settings.

These limitations highlight important considerations for the use of AI in interpreting radiological descriptions and underscore the necessity for the rigorous validation and continuous improvement of AI systems in clinical settings.

## 5. Conclusions

Our study underscores the diverse capabilities of AI models in scoliosis classification and therapeutic recommendation. Although models such as ChatGPT 4, Copilot, and PopAi demonstrated high alignment with clinical standards because of extensive and diverse training data, others, like PMC-LLaMA 13B, showed significant deviations, particularly in expanding the range for moderate scoliosis, which could delay therapeutic interventions. This variability highlights the importance of continuous evaluation and recalibration, emphasizing the need for incorporating more diverse and representative training datasets. Advanced transformer architectures used in models like ChatGPT 4 and Copilot play a crucial role in their superior performances, enabling them to capture complex patterns and relationships in data more effectively than simpler models. However, all the models exhibited challenges in maintaining consistency with established medical standards in their responses, indicating the need for improved autonomy in decision-making based on medical knowledge. Ethical considerations, bias mitigation, and the scalability of AI solutions are also critical, as the deployment of AI in clinical settings involves regulatory approvals and ensuring equitable and unbiased healthcare recommendations. These findings point to the necessity of the rigorous validation and continuous improvement of AI systems to enhance their clinical reliability and relevance, ensuring they can effectively support healthcare professionals in diagnostic and therapeutic decision-making processes.

## Figures and Tables

**Table 1:** This table presents the parameter settings of the PMC-LLaMA 13B model.

Parameter	Value
max_new_tokens	512
temperature	0.7
top_p	0.1
min_p	0
top_k	40
repetition_penalty	1.18
presence_penalty	0
frequency_penalty	0
repetition_penalty_range	1024
typical_p	1
tfs	1
top_a	0
epsilon_cutoff	0
eta_cutoff	0
guidance_scale	1
minostat_mode	0
minostat_tau	5
minostat_eta	0.1
smoothing_factor	0
smoothing_curve	1
dynamic_temperature	do_sample
num_beams	1
max_tokens/prompt	2048

**Table 2:** Prevalence and severity distribution of scoliosis in pediatric radiographs.

Characteristic	*N*	Distribution
Cobb angle	72	29.50 (16.75, 54.25) ^a^
Severity of disease ^1^:	72	
mild		25.00 (34.72%) ^b^
moderate		19.00 (26.39%) ^b^
severe		28.00 (38.89%) ^b^

^a^ *Mdn* (*Q1*, *Q3*); ^b^ *n* (%) ^1^ with 100% agreement between raters. *N*—sample size; *n*—group size; *Mdn*—median; *Q1*—the first quartile (25%); *Q3*—the third quartile (75%).

**Table 3:** AI systems’ classifications and treatment recommendations for scoliosis based on Cobb angle ranges.

AI System	Severity of Disease			Treatment Modality		
	Mild	Moderate	Severe	Monitoring	Bracing	Surgery
Reference level	<20	20–40	>40	<20	20–40	>40
ChatGPT 4	<20	20–40	>40	<20	20–40	>40
Copilot	<20	20–40	>40	<20	20–40	>40
Gemini	10–25	25–40	>40	Did not undertake classification		
Gemini Advanced	10–25	25–45	>45	Did not undertake classification		
PopAi	<20	20–40	>40	<20	20–40	>40
You Research	10–24	25–39	≥40	10–24	25–45	>45
You GPT 4	10–20	21–40	>40	10–24	25–39	≥40
You Genius	10–24	25–39	≥40	10–24	25–45	>45
Claude 3 Opus	10–20	21–40	>40	<25	25–45	>45
Claude 3 Sonnet	10–25	25–40	≥40	<20	20–50	>50
PMC-LLaMA 13B	10–20	20–50	>50	10–20	20–50	>50

**Table 4:** Comparative performances of AI systems in scoliosis severity and treatment modality classifications.

AI System	Overall Sample				Category-Wise											
					Severity Classification						Treatment Classification					
	*SC*	*ST*	*CC*	*CT*	Mild		Moderate		Severe		Monitoring		Bracing		Surgery	
					*SC*	*ST*	*SC*	*ST*	*SC*	*ST*	*CC*	*CT*	*CC*	*CT*	*CC*	*CT*
Chat GPT 4	1.00	1.00	1.00	1.00	1.00	1.00	1.00	1.00	1.00	1.00	1.00	1.00	1.00	1.00	1.00	1.00
Copilot	1.00	1.00	1.00	1.00	1.00	1.00	1.00	1.00	1.00	1.00	1.00	1.00	1.00	1.00	1.00	1.00
Gemini	n/a	n/a	n/a	n/a	n/a	n/a	n/a	n/a	n/a	n/a	n/a	n/a	n/a	n/a	n/a	n/a
Gemini Advanced	0.77	0.68	n/a	n/a	0.79	0.74	0.60	0.43	0.88	0.82	n/a	n/a	n/a	n/a	n/a	n/a
PopAi	1.00	1.00	0.83	0.83	1.00	1.00	1.00	1.00	1.00	1.00	0.66	0.66	0.77	0.77	1.00	1.00
You Research	n/a	n/a	n/a	n/a	n/a	n/a	n/a	n/a	n/a	n/a	n/a	n/a	n/a	n/a	n/a	n/a
You GPT 4	0.67	0.67	n/a	n/a	0.77	0.77	0.49	0.49	0.83	0.83	n/a	n/a	n/a	n/a	n/a	n/a
You Genius	n/a	n/a	0.78	n/a	n/a	n/a	n/a	n/a	n/a	n/a	0.58	n/a	0.74	n/a	0.97	n/a
Claude 3 Opus	0.96	0.96	0.77	0.98	0.94	0.94	0.93	0.93	1.00	1.00	0.60	0.97	0.79	0.96	0.88	1.00
Claude 3 Sonnet	0.85	0.81	0.50	0.58	0.91	0.73	0.85	0.91	0.89	0.89	0.45	0.67	0.67	0.58	0.38	0.49
PMC LLaMA 13B	0.55	0.49	0.37	0.24	0.68	0.68	0.33	0.30	0.66	0.53	0.13 *	0.13 *	0.18 *	0.02 *	0.74	0.57

*Note: SC*—agreement between reference evaluation and AI system for scoliosis severity classification; *ST*—agreement between AI-estimated severity intervals and actual estimates based on the individual Cobb angles; *CC*—agreement between reference evaluation and AI system for treatment modality; *CT*—agreement between AI-estimated treatment modality intervals and actual estimates based on the individual Cobb angles; n/a—not applicable (AI system did not undertake classification); *—non-significant agreement (*p* > 0.050).

**Table 5:** Comparative analysis of AI systems: confusion matrix and severity classification performance metrics.

AI System	Overall Accuracy	Severity Class	Sensitivity	Specificity	Positive Predictive Value	Negative Predictive Value	Precision	Recall	F1	Prevalence	Detection Rate	Detection Prevalence	Balanced Accuracy
ChatGPT 4; Copilot; PopAi	1.00	Mild	1.00	1.00	1.00	1.00	1.00	1.00	1.00	0.35	0.35	0.35	1.00
		Moderate	1.00	1.00	1.00	1.00	1.00	1.00	1.00	0.26	0.26	0.26	1.00
		Severe	1.00	1.00	1.00	1.00	1.00	1.00	1.00	0.39	0.39	0.39	1.00
Gemini Advanced	0.85	Mild	0.92	0.89	0.82	0.95	0.82	0.92	0.87	0.35	0.32	0.39	0.91
		Moderate	0.68	0.91	0.72	0.89	0.72	0.68	0.70	0.26	0.18	0.25	0.79
		Severe	0.89	0.98	0.96	0.93	0.96	0.89	0.92	0.39	0.35	0.36	0.94
You GPT 4	0.82	Mild	0.75	1.00	1.00	0.88	1.00	0.75	0.86	0.35	0.26	0.26	0.88
		Moderate	0.81	0.83	0.59	0.93	0.59	0.81	0.68	0.24	0.19	0.32	0.82
		Severe	0.89	0.93	0.89	0.93	0.89	0.89	0.89	0.41	0.37	0.41	0.91
Claude 3 Opus	0.97	Mild	1.00	0.96	0.93	1.00	0.93	1.00	0.96	0.35	0.35	0.38	0.98
		Moderate	0.90	1.00	1.00	0.96	1.00	0.89	0.94	0.26	0.24	0.24	0.95
		Severe	1.00	1.00	1.00	1.00	1.00	1.00	1.00	0.39	0.39	0.39	1.00
Claude 3 Sonnet	0.94	Mild	0.92	1.00	1.00	0.96	1.00	0.92	0.96	0.35	0.32	0.32	0.96
		Moderate	0.88	1.00	1.00	0.96	1.00	0.88	0.94	0.25	0.22	0.22	0.94
		Severe	1.00	0.90	0.88	1.00	0.88	1.00	0.93	0.41	0.41	0.46	0.95
PMC-LLaMa 13B	0.73	Mild	0.88	0.81	0.73	0.92	0.73	0.88	0.80	0.37	0.32	0.44	0.85
		Moderate	0.47	0.84	0.53	0.80	0.53	0.47	0.50	0.27	0.13	0.25	0.66
		Severe	0.79	0.95	0.90	0.89	0.90	0.79	0.84	0.35	0.28	0.31	0.87

**Table 6:** Performance metrics and confusion matrix data for comparative analysis of AI systems in treatment modality classification.

AI System	Overall Accuracy	Severity Class	Sensitivity	Specificity	Positive Predictive Value	Negative Predictive Value	Precision	Recall	F1	Prevalence	Detection Rate	Detection Prevalence	Balanced Accuracy
ChatGPT 4; Copilot	1.00	Monitoring	1.00	1.00	1.00	1.00	1.00	1.00	1.00	0.35	0.35	0.35	1.00
		Bracing	1.00	1.00	1.00	1.00	1.00	1.00	1.00	0.26	0.26	0.26	1.00
		Surgery	1.00	1.00	1.00	1.00	1.00	1.00	1.00	0.39	0.39	0.39	1.00
PopAi	0.89	Monitoring	1.00	0.83	0.76	1.00	0.76	1.00	0.86	0.35	0.35	0.46	0.91
		Bracing	0.58	1.00	1.00	0.87	1.00	0.58	0.73	0.26	0.15	0.15	0.79
		Surgery	1.00	1.00	1.00	1.00	1.00	1.00	1.00	0.39	0.39	0.39	1.00
You Genius	0.86	Monitoring	1.00	0.81	0.74	1.00	0.74	1.00	0.85	0.35	0.35	0.47	0.90
		Bracing	0.52	0.98	0.91	0.85	0.91	0.53	0.67	0.26	0.14	0.15	0.75
		Surgery	0.96	1.00	1.00	0.98	1.00	0.96	0.98	0.39	0.38	0.38	0.98
Claude 3 Opus	0.85	Monitoring	0.96	0.87	0.80	0.98	0.80	0.96	0.87	0.35	0.33	0.42	0.92
		Bracing	0.68	0.91	0.72	0.89	0.72	0.68	0.70	0.26	0.18	0.25	0.79
		Surgery	0.86	1.00	1.00	0.92	1.00	0.86	0.92	0.39	0.33	0.33	0.93
Claude 3 Sonnet	0.67	Monitoring	0.80	0.87	0.77	0.89	0.77	0.80	0.78	0.35	0.28	0.36	0.84
		Bracing	0.74	0.77	0.54	0.89	0.54	0.74	0.62	0.26	0.19	0.36	0.76
		Surgery	0.50	0.86	0.70	0.73	0.70	0.50	0.58	0.39	0.19	0.28	0.68
PMC-LLaMa 13B	0.59	Monitoring	0.24	0.91	0.60	0.69	0.60	0.24	0.34	0.35	0.08	0.14	0.58
		Bracing	0.63	0.62	0.38	0.82	0.38	0.63	0.47	0.27	0.17	0.45	0.62
		Surgery	0.89	0.89	0.83	0.93	0.83	0.89	0.86	0.38	0.34	0.41	0.89

## Data Availability

The data are contained within the article.
